# Detection of size of manufactured sand particles based on digital image processing

**DOI:** 10.1371/journal.pone.0206135

**Published:** 2018-12-14

**Authors:** Jianhong Yang, Wen Yu, Huai-ying Fang, Xiao-yu Huang, Si-jia Chen

**Affiliations:** 1 Department of Key Laboratory of Process Monitoring and System Optimization for Mechanical and Electrical Equipment (Huaqiao University), Fujian Province University, Huaqiao University, Xiamen, Fujian Province, China; 2 Department of Radiation Oncology, Xiamen Cancer Hospital, First Affiliated Hospital of Xiamen University, Xiamen, Fujian Province, China; University of Sheffield, UNITED KINGDOM

## Abstract

The size distribution of manufactured sand particles has a significant influence on the quality of concrete. To overcome the shortcomings of the traditional vibration-sieving method, a manufactured sand casting/dispersing system was developed, based on the characteristics of the sand particle contours (as determined by backlit image acquisition) and an extraction mechanism. Algorithms for eliminating particles from the image that had be repeatedly captured, as well as for identifying incomplete particles at the boundaries of the image, granular contour segmentation, and the determination of an equivalent particle size, are studied. The hardware and software for the image-based detection device were developed. A particle size repeatability experiment was carried out on the single-grade sands, grading the size fractions of the manufactured sand over a range of 0.6–4.75 mm. A method of particle-size correction is proposed to compensate for the difference in the results obtained by the image-based method and those obtained by the sieving method. The experimental results show that the maximum repeatability error of single-grade fractions is 3.46% and the grading size fraction is 0.51%. After the correction of the image method, the error between the grading size fractions obtained by the two methods was reduced from 7.22%, 6.10% and 5% to 1.47%, 1.65%, and 3.23%, respectively. The accuracy of the particle-size detection can thus satisfy real-world measuring requirements.

## Introduction

Differences in processing technology lead to fluctuations in the quality of manufactured sand. Therefore, to ensure the quality of manufactured sand, there is a need for a means of monitoring the grain sizes in line with national standards (Test Methods of Aggregate for Highway Engineering (JTG E42-2005))[1]. The vibrating-sieve method is a well-known method of determining the size of an aggregate, but the sieving process itself tends to destroy the gravel and affect the original grain size. Furthermore, the screening accuracy is affected by the grain shape, adversely affecting the accuracy of the test results. An image-based system, however, can accurately detect the granularity of manufactured sand and does not incur any destruction of the particles.

In 1998, Fernlund found that the size of an aggregate as determined by the sieving method was greatly affected by the particle shape. In contrast, the image-based method can acquire a more realistic particle axial size, with the results being closer to those obtained manually [2]. The VDG-40 Videograder system developed by Browne et al. in 2001 assumed that each particle was an ellipsoid and calculated the third diameter of a particle from the two diameters acquired from a two-dimensional image of the particle [3]. In 2012, Kumara et al. discussed the advantages of particle-size measurement using an image-based method. Using the equivalent ellipse short axis as the particle size, the results obtained with the image-based method were greater than those obtained with the sieving method. The particle size determined by the image-based analysis should be appropriately adjusted to fit the gradation curve of the image-based and sieving methods, such that the fitting coefficient can be calculated [4]. FIVE years later, they proposed a method for obtaining a particle-based gradation curve by obtaining a volume-based gradation curve from a 2D image, Analyzed different ways in which particles pass through the sieve, the correction factor between the sieve methods and the image method is calculated.[5] In 2014, Sulaiman et al. applied automatic image-processing technology to this problem. Using the automatic threshold for image binarization, the morphological closed operation and watershed algorithm were used to remove the grain texture and separate the particles. The particle-size distribution characteristics of river-bed gravel were analyzed by using the equivalent elliptical short axis as the particle-size [6]. Hamzeloo, E et al. detected the image of the stacked ore with 5mm and above in diameter on the conveyor belt. It was found that the diameter of the largest inscribed circle of the particle was taken as the diameter, and the volume of the sphere of the diameter was taken as the volume, the measured particle size results and the sieving method have less error. [7]Junxing Zheng et al. presented a particle size characterization method based on stereophotography. The thickness of the particles can be accurately obtained by this method. After obtaining the thickness of every particle from this method and the length and width from traditional 2D single images, an ellipsoidal particle model is used to approximate the three particle dimensions. The equivalent sieve opening size is then determined by mathematically fitting the ellipsoidal particle to a square sieve opening.[8] Cheng et al. proposed an online visual inspection method is proposed for the size and shape of molecular sieves. The main measured particle size is between 1mm and 3mm (but not for mechanical sand).[9] In 2016, Okpeafoh et al. combined the image method with the measured chord length data to obtain a reliable particle-size distribution and aspect ratio [10]. Yang J proposed an online detection system for aggregate sizes and shapes based on digital image processing. In contrast to traditional methods, this system can be installed in a production field to monitor aggregate quality in real time.[11]In 2002, Miao et al. developed an image-based particle measurement software package, capable of detecting the size of pulverized coal particles, which realized automatic correction, automatic segmentation, automatic detection, and automatic storage of the particles identified in a single image [12]. In 2014, Shi manually examined aggregate particles against a backlight and devised a system based on Labview that could identify a variety of features of each particle [13]. In 2014, Yan processed asphalt mixture slices, identifying a coarse aggregate gradation with a particle-size range of 2.36–19 mm. The aggregate with a particle size of between 2.36 and 4.75 mm was multiplied by a correction factor of 2.1 so that the calculated gradation was close to the design gradation curve [14].

However, some of these methods require particles to be uniformly spread out on a plane by hand. Particles were measured in a picture, and the entire measurement system was not established. For particle size, the particle size range of the study is more than 1mm, and no particles below 1mm have been studied.

The present study addressed a test system that was designed to measure the granularity of sand. The optimal characterization parameters of the manufactured sand were studied experimentally. The results of repeated measurements showed that the system offers good reliability. The results obtained by the developed image-based method and vibration-screening method are compared and corrected. The modified sand particle-size measurement accuracy ultimately satisfies the engineering monitoring requirements.

For the present study, the particle-size range of the manufactured sand was set to between 0.6 and 4.75 mm. According to the JTG E42-2005 standard [1], sand is divided into several single grades. In this standard, the screen sizes are set to 0.6 mm, 1.18 mm, 2.36 mm, and 4.75mm.

## Materials and methods

### Development of particle-size testing system

#### System hardware

**Fig 1:**
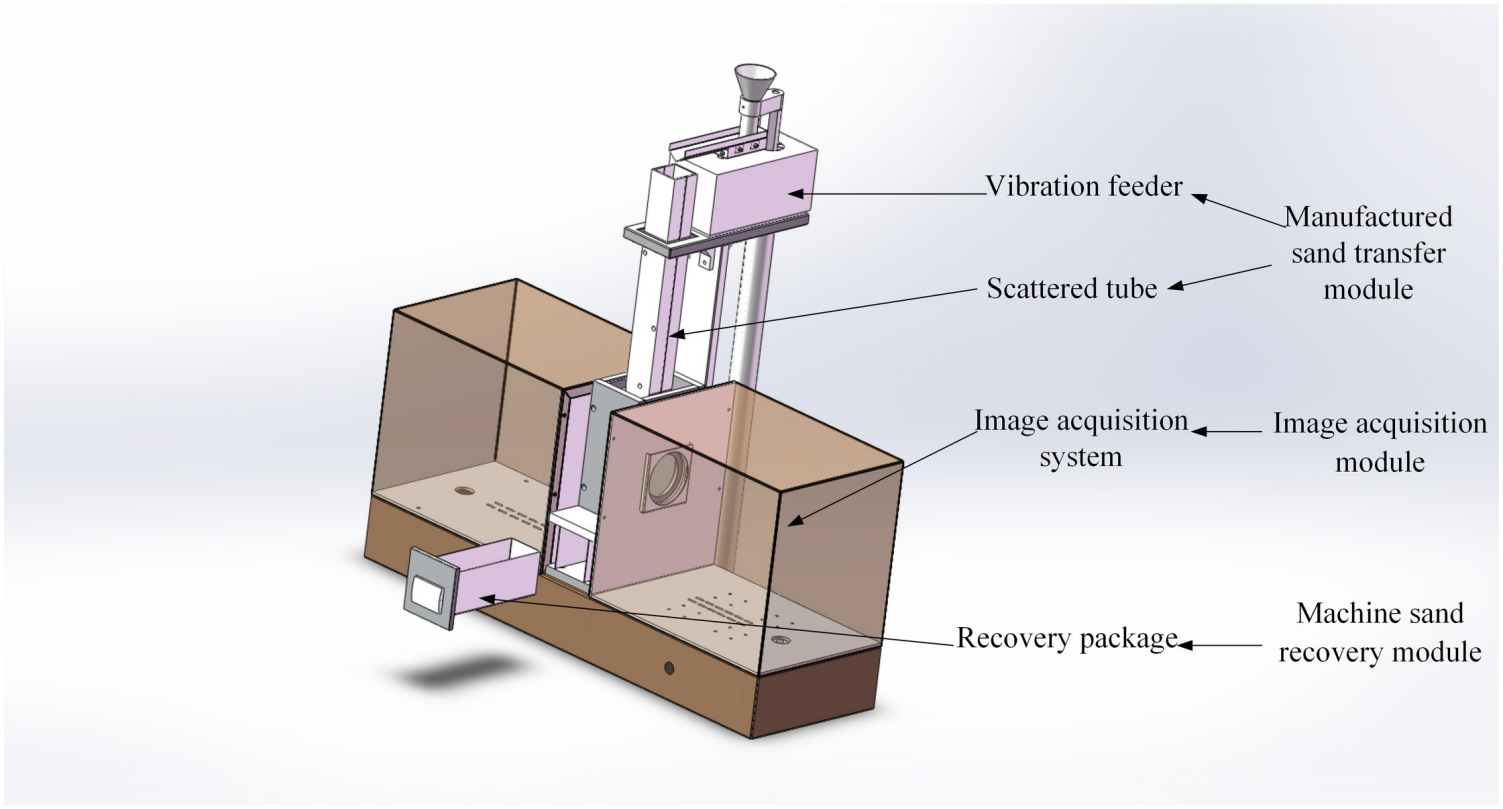
Particle-size detection system.

The particle-size range of the target measurement is greater, but the overall grain size is smaller. The effect of gravity and external forces (such as vibration) will lead to the segregation of the sand. Any kind of stacking would make it difficult to capture a complete profile even of the surface of the particles, thus increasing the complexity of the image processing. In contrast, a mechanism capable of dispersing the sand particles would prevent any segregation or stacking, and would enable the capture of a more complete profile of the particles. The developed measurement system hardware consists of a sand-transfer module, image-acquisition module, and sand-recovery module. The sand-transfer module consists of vibration feeder and scattered tube, the vibrating feed conveys the manufactured sand continuously to the scattered tube, and the particles achieve a uniform dispersion effect. Particles passing through the dispersion tube enter the image-acquisition module, this module consists of industrial camera and lens, and LED(Light-Emitting Diode Light) backlight. Finally, the detected manufactured sand enters the sand-recovery module for recycling. The overall structure of the measurement system is shown in Fig 1.

The main parameters of the device are listed in Table 1.

**Table 1:** Device parameters.

Device	Main parameters
Vibration feeder	Electromagnetic drive, stroke of 5 mm, 0–24 V voltage regulation, maximum amplitude of 1.5 mm, maximum frequency of 3000 rpm, active power 5 W.
Industrial camera	Resolution of 640 × 480 pixels, visual field size of 80 × 60 mm^2^, pixel accuracy of 1/8 mm. To attain a frame rate of 120 frames/s, the minimum exposure time is set to 0.1 μs.
Lens	Focal length of 8 mm, aperture of 1:1.4–16, distortion of less than 0.5%.
LED backlight	Size of 150 × 150 mm^2^, voltage range of 0–24 V, with 256 levels of brightness.

Stability tests of the vibration feeder were carried out. The sand conveyance was tested by measuring at the feeder at a constant sampling frequency but different vibration frequencies. We analyzed the linear correlation with the number of samplings to determine the stability. The samples were selected as single-grade 0.6–1.18 mm, 1.18–2.36 mm, 2.36–4.75 mm, as well as a grading of 0.6–4.75 mm. The raw material was limestone. For each of the single grades, a 50-g sample was acquired. The frequency of the vibratory feeder was set to 70 Hz, 80 Hz, and 90 Hz. The sampling period of the weighing device was 200 ms. A linear correlation analysis was performed on the measured data, with the correlation being calculated according to Eq (1):

$$r=\frac{\sum_{i=1}^{n}\left(x_{i}-\overset{-}{x}\right)\left(y_{i}-\overset{-}{y}\right)}{\sqrt{\sum_{i=1}^{n}{\left(x_{i}-\overset{-}{x}\right)}^{2}\times {\left(y_{i}-\overset{-}{y}\right)}^{2}}}$$ (1)

where, r is the linear correlation coefficient, x is the sampling count, and y is the transmission quality.

The above experiment was carried out on the single-stage material of the sieved sand. The results are listed in Table 2.

**Table 2:** Linear correlation coefficients for different particle sizes/frequencies.

Particle-size distribution (mm)	Linear correlation coefficient of different particle size at different frequencies		
	75	80	90
0.6–1.18	0.995	0.980	0.951
1.18–2.36	0.994	0.986	0.972
2.36–4.75	0.993	0.986	0.961
0.6–4.75	0.990	0.972	0.958

As the vibration frequency falls, the linear correlation coefficient increases, such that the vibration feeder stability improves. The difference between the linear correlation coefficient r is 0.044, so the change in the vibration frequency has little effect on the stability of the vibration feeder.

#### Software system

The particle-size detection software consists of: image-acquisition module, and image-processing module, the latter being responsible for image preprocessing, image segmentation, and the removal of repeatedly captured particles.

#### Image preprocessing

The preprocessing consists mainly of grayscale processing and filtering. Because of the rough burrs left at the edges of the particles after grayscale processing, the results of some particle binarization exhibit very rough edges. To avoid the grain edge fracture and truly restore the shape contours of the particles, a Gaussian filter is used to give the particles a smooth boundary.

#### Image segmentation

Image segmentation is divided into region, boundary and threshold segmentation. In the present study, the maximum interclass variance method proposed by Otsu in 1979 was used for the threshold segmentation [15]. We assumed that the threshold, T, divides each pixel into two categories according to the gray value. The C_0_ class contains pixels with a grayscale range of [0, 1, …, z], and the probability of each gray value for the C_0_ class is W_0_, as defined by Eq (2):

$$w_{0}=\sum_{i=0}^{z}P_{i}$$ (2)

where, w_0_ is the probability of each gray value of the C_0_ class and Pi is the probability of each gray value.

Then, the expected mean of the C_0_ class is μ_0_, as given by Eq (3):

$$\mu_{0}=\sum_{i=0}^{z}iP_{i}/w_{0}$$ (3)

where, μ_0_ is the expected mean of the C_0_ class.

The C_1_ class contains pixels with a grayscale range of [z+1, z+2, …, k-1], and the probability of each gray value of the C_1_ class is w_1_, as defined by Eq (4):

$$w_{1}=\sum_{i=z+1}^{k-1}P_{i}$$ (4)

where, w_1_ is the probability of each gray value of the C_1_ class and Pi is the probability of each gray value.

Then, the expected mean of the C_1_ class is μ_1_, as given by Eq (5):

$$\mu_{1}=\sum_{i=z+1}^{k-1}iP_{i}/w_{1}$$ (5)

where, μ_1_ is the expected mean of class C_1_.

The total gray scale of the image is μ, and the defined inner variance is σ, as given by Eqs (6) and (7):

$$\mu ={\text{w}}_{0}\mu_{0}+w_{1}\mu_{1}$$ (6)

σ=[w0(μ−0μ)2−w1(μ1−μ)2] (7)

In practical applications, any calculation based on the above formula would be extremely costly in terms of time and resources, so for practical applications, the optimal threshold T is determined using Eq (8):

$$T=\text{max}\left[{\text{w}}_{0}w_{1}{\left(\mu_{0}-\mu_{1}\right)}^{2}\right]$$ (8)

**Fig 2:**
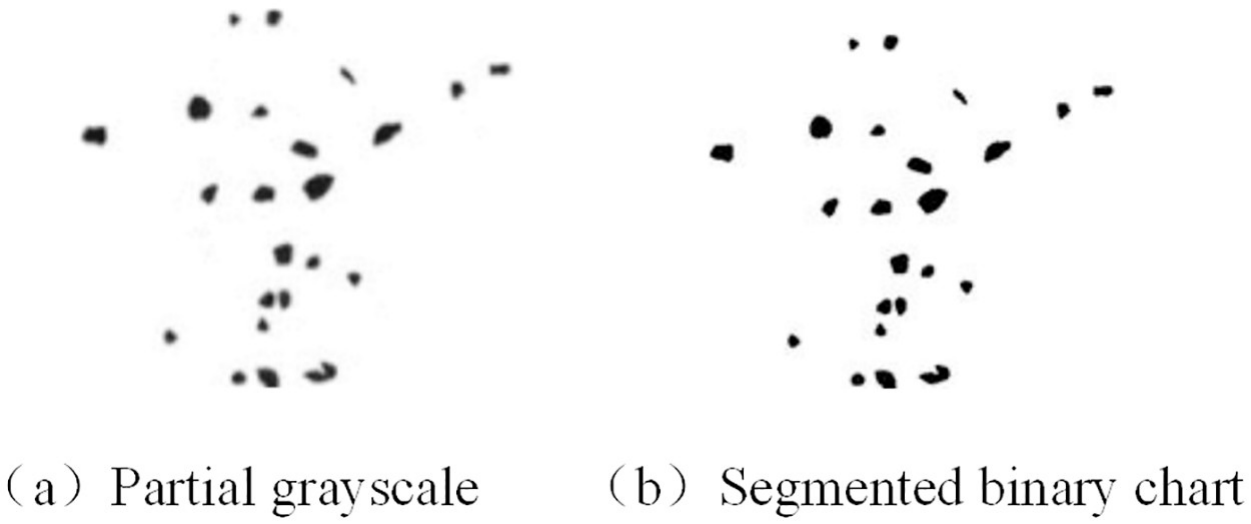
Split effect.

The maximum interclass variance method is used to segment the filtered sand image shown in Fig 2(a). The results are shown in Fig 2(b). The background and the target particles are accurately separated, and each contour is accurately presented in the binary image.

#### Removal of repeatedly captured particles

**Fig 3:**
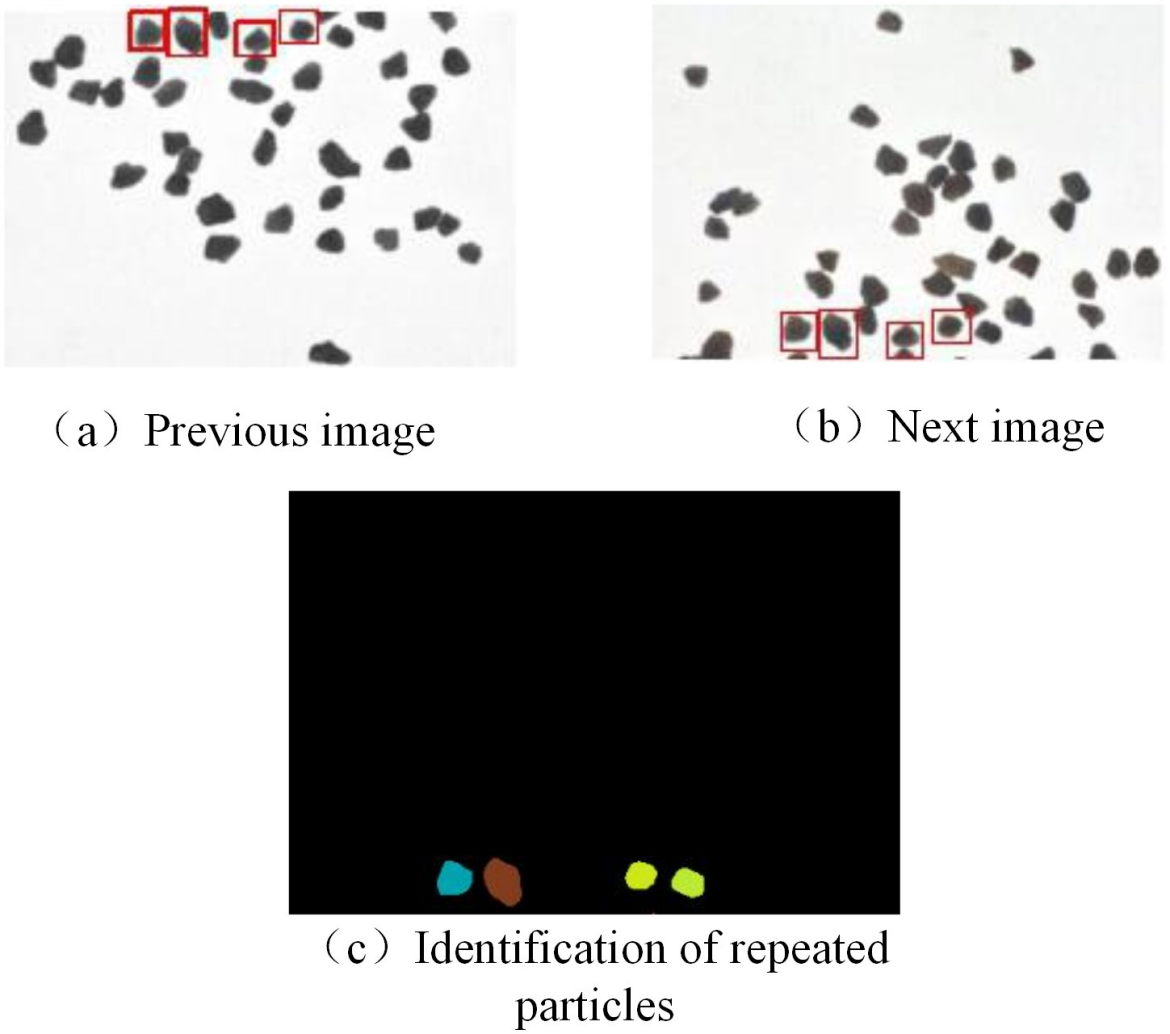
Repeated particle capture.

In the image-acquisition stage, the image acquisition rate is related to the frame rate. When the frame rate is too high, a given particle will appear in consecutive images; an example of the same particle appearing in adjacent images is shown in Fig 3(a) and 3(b). To eliminate this repeated capture of particles, the repeated sand is identified by the Hu moment. Two contours, A and B, are defined. Metric I_1_(A,B) can be used to compare A and B, as given by Eqs (9)–(11):

$$I_{1}(A,B)=\sum_{\text{i}=1}^{7}\left|\frac{1}{{\text{m}}_{i}^{A}}-\frac{1}{{\text{m}}_{i}^{B}}\right|$$ (9)

$${\text{m}}_{i}^{A}=sign(h_{i}^{A})•\text{log}\left|h_{i}^{A}\right|$$ (10)

$${\text{m}}_{i}^{B}=sign(h_{i}^{B})•\text{log}\left|h_{i}^{B}\right|$$ (11)

where, |h_i_^A^| and |h_i_^B^| are the invariant distances of A and B, respectively. When the threshold is equal to 0.008, metric standards are the most sensitive to contour differences between the particles and can identify repeatedly captured particles. The results are shown in Fig 3(c).

#### Removal of incomplete particles at the boundary

**Fig 4:**
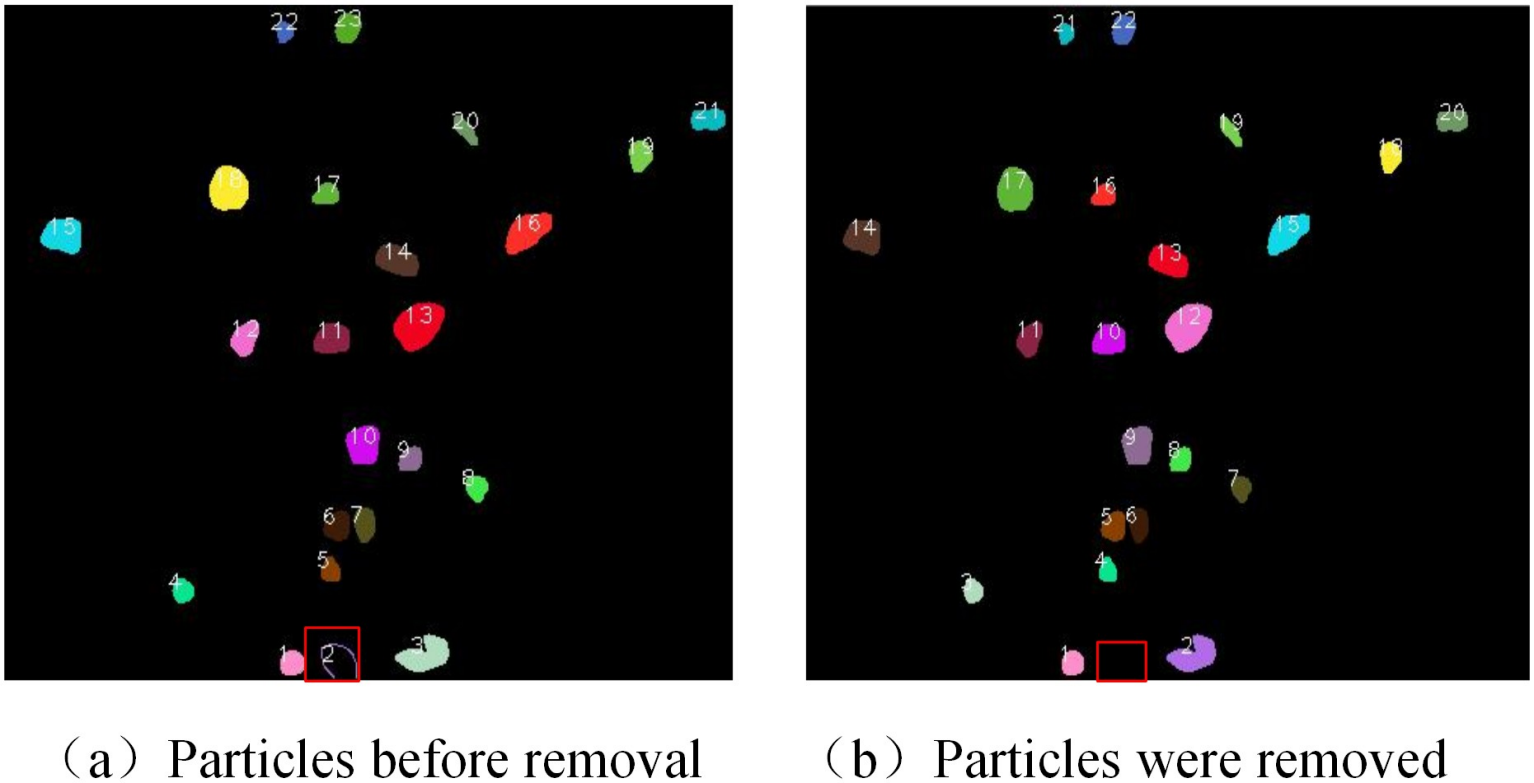
Removal of incomplete particles at boundary.

There will always be incomplete particles at the image boundary. If these particles were to be incorporated into the calculations, the particle-size and grain distribution would be affected. Therefore, it is necessary to remove these incomplete particles. The particles that are connected to the boundary exhibit a non-closed curve upon edge detection, as shown in Fig 4 for particles #2 in the red box. The total area and perimeter of the 23 particles in the figure are calculated, and the perimeter-to-area ratio obtained, as shown in Table 3.

**Table 3:** Circumference ratio of grain area.

Particle number	Area	Circumference	Perimeter-to-area ratio
1	180	50	3.60
2	10	120	0.08
3	446	104	4.29
4	148	47	3.15
5	133	45	2.96
6	228	60	3.80
7	216	58	3.72
8	161	49	3.29
9	165	51	3.24
10	391	77	5.08
11	330	70	4.71
12	259	64	4.05
13	656	101	6.50
14	383	80	4.79
15	420	81	5.19
16	464	92	5.04
17	160	50	3.20
18	528	87	6.07
19	206	56	3.68
20	136	52	2.62
21	226	63	3.59
22	102	40	2.55
23	223	58	3.84

The area circumference ratio of particle 2 in Table 3 is 0.08, which is much smaller than that of the other non-boundary particles. Among the complete particles, the minimum area perimeter ratio appears for particle 22, for which the value is 2.55. It is possible to select an area circumference ratio of less than 1 as a condition for judging whether the boundary particles are present. When the ratio of a particle is less than 1, it is deemed to be a boundary particle, and is then removed from the contour sequence. The result of removing the incomplete particles in the red box in Fig 4(a) is shown in Fig 4(b).

#### Adhered particle contour segmentation

**Fig 5:**
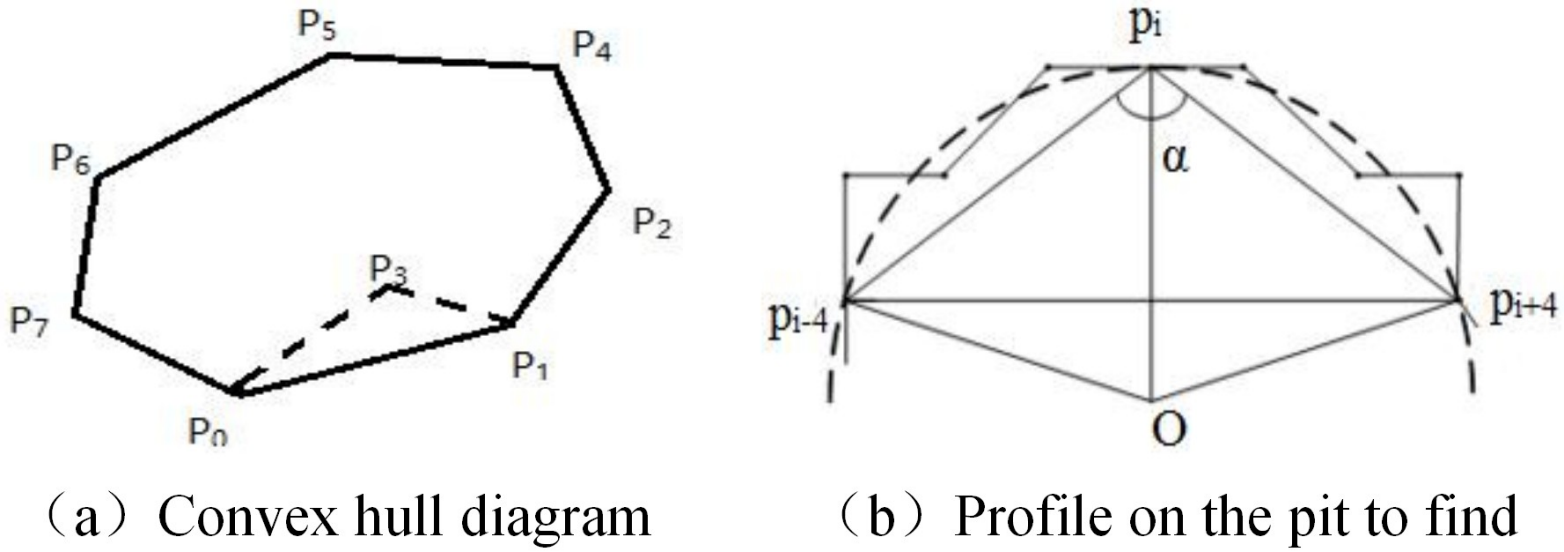
Concave point detection.

Even when using a vibratory dispersion device, when the manufactured sand grade is changed, or if the water content increases, there will always be particle adhesion. Multiple particles will therefore be in contact with each other in the captured image, and subsequently will be binarized as a single particle, which affects the statistical results. The premise for separating such adhered particles is to determine the adhesive particle, which can be determined by counting the convex hulls. A convex hull is the smallest convex polygon that encloses a boundary point. Assuming that there are 8 points, P0–P7, on the plane and some convex polygons, so that the polygon surrounds all the points, the polygon can be called a “convex hull,” as shown in Fig 5(a). The ratio of the actual area of the particles to the convex hull area is calculated. As the difference between the actual area and that of the convex hulls increases, it becomes more likely that the particle has been formed by the adhesion of particles. When the threshold is 0.9, it can be judged as being optimum, but when the ratio of the actual area to that of the convex hulls is less than 0.9, it is judged as being made up of adhered particles. When the ratio is more than or equal to 0.9, it is judged to be a single particle. To determine the contour of the adhered particles, based on the sharpness of the contour, we can find the junction point of particles, which is a concave point. Fig 5(b) is a schematic diagram of the concave point.

The support area contains the contour point pi which is the center, the four points of pi and four posterior points of pi, with the angle α of the three points giving the supporting foot. Here, α is the distance between the two supporting points divided by the sum of the length of the two support lines which can be used to approximate the support angle. As the support angle increases, it is less likely to be a concave point. The sharpness is defined by Eq (12):

$$\text{sharp}=1-\alpha =1-\frac{\left|p_{i-4}p_{i+4}\right|}{\left|p_{i}p_{i-4}\right|+\left|p_{i}p_{i+4}\right|}$$ (12)

**Fig 6:**
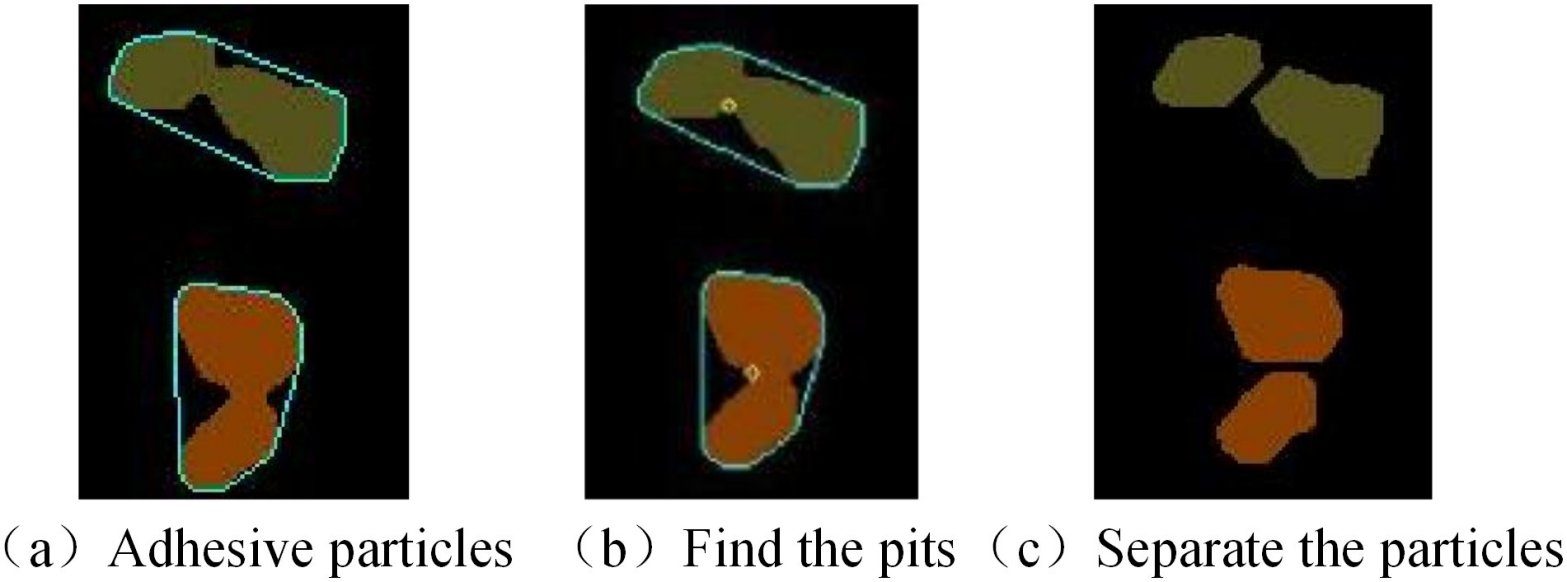
Separation of adhered particles based on pits.

The above formula is used to indicate the sharpness of the support angle. As the value of α decreases, the shape becomes larger and the sharpness better. To calculate the sharpness of each point on the contour sharpness (pi), set a threshold T such that sharp (pi) > T, and pi is the concave point to find. Fig 6(b) shows the identification of the concave points of the adhered particles. Two adjacent concave points are connected to give a dividing line. Starting from one concave point, the dividing line passes through the interior of the particle to the other side of the particle, thus dividing the connected particles. The separated adhered particles are shown in Fig 6.

#### Study of characterization parameters of granularity

The granularity of the sand was measured by the sieving method in the interval [ai, ai+1](unit: mm), with the mass proportion of the particles, g, being given by Eq (13).

$$g=\frac{m}{M}=\frac{\rho V_{m}}{\rho V_{M}}=\frac{V_{m}}{V_{M}}$$ (13)

where m is the mass of the sand in the interval, M is the total mass of the total sand of a given particle size, ρ is the density of the sand, Vm is the volume of the sand in the particle-size interval [ai, ai + 1], and VM is the total volume of sand. Thus, there are two parameters that will affect the particle-size distribution test results, that is, the size of the sand D and the volume of sand V.

**Fig 7:**
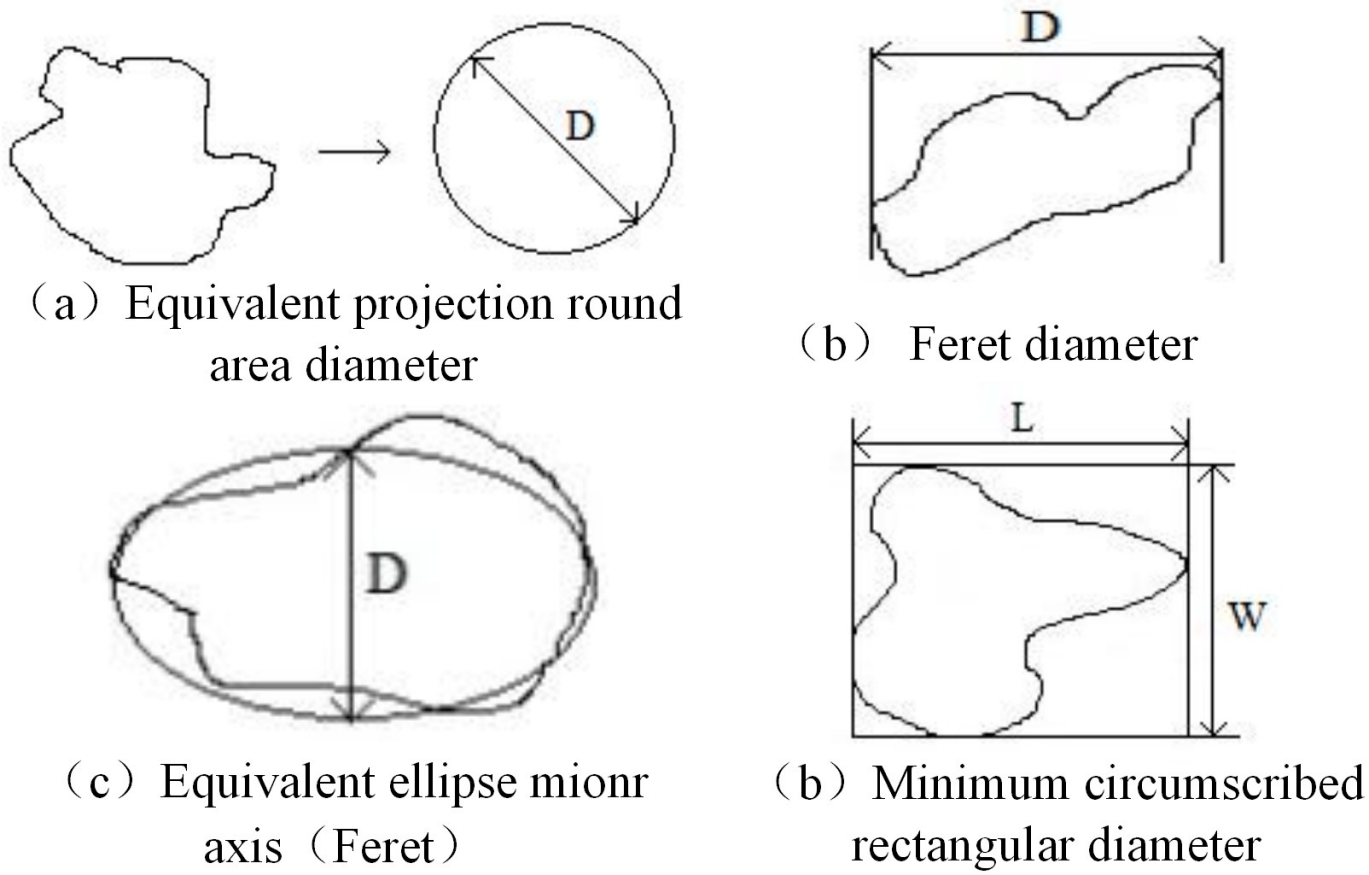
Characterization algorithm particle size.

When using the image method to measure the granularity of the sand, it is necessary to select the appropriate equivalent particle size for the sand particles in the image. Only the spherical particles and the circular projection shape can be assigned a numerical value, that is, the diameter is used to describe its size. Meanwhile, other irregular non-spherical particles need to use some other parameters to represent the particle size. The commonly used characterization algorithm particle size involves the following:

1. Equivalent projection round area diameter: When the projected area S of a manufactured sand in the image is equal to the area of a circle of diameter R, and the diameter of the circle becomes the equivalent projected circle area diameter of the sand, D, as shown in Fig 7(a).
2. Feret diameter: two parallel lines tangent to the projection profile of the manufactured sand in the image. The distance between the parallel lines is the Feret diameter of the manufactured sand. As shown in Fig 7(b), the maximum (minimum) Feret trail (the maximum (minimum) distance between two parallel lines tangential to the projection profile of the sand). The equivalent ellipse Feret short diameter is the minor axis of the ellipse with the same area as the working sand and a long axis that is equal to the maximum Feret diameter of the manufactured sand. As shown in Fig 7(c), the maximum Feret diameter is the long axis, and D is the minor axis of the ellipse with an area equal to that of the particle.
3. Minimum circumscribed rectangular diameter: the smallest circumscribed rectangle that can surround the entire sand particle system, with the largest horizontal, vertical, minimum horizontal, and vertical coordinates of the manufactured sand projection image used to determine the boundary, as shown in Fig 7(d), where L is the length of the smallest circumscribed rectangle and W is the width of that rectangle.

When using sieve screening, a small proportion of the sand is left on the screen due to clogging or a lack of screening time. Therefore, for a sieve of a given mesh size, there will be some manufactured sand that is smaller than the mesh size. This will result in the manufactured sand being classed as a smaller grade than that determined using the image-based method. Watano et al. studied the image-based method and used the equivalent elliptical Feret short diameter as the characterization parameter of the equivalent particle size. [16] The equivalent ellipse minor axis (Feret) is used as the characterization parameter of the equivalent particle size. Because we want to calculate the mass ratio, we also need to determine the equivalent volume of the particle, with the product of the projection area and the equivalent ellipse minor axis (Feret) giving the most suitable equivalent volume.

## Results and discussion

### Experimental tests and analysis of results

#### Particle-size repeatability test

In the present study, the manufactured sand particle-size detection system was used for single-stage materials and to grade ingredients as a repeatability test. The single-stage materials were 0.6–1.18 mm, 1.18–2.36 mm, and 2.36–4.75 mm, respectively. The manufactured sand was composed of granite form Fujian province. According to “Test Methods of Aggregate for Highway Engineering” (JTG E42-2005)[1], for the AC-5 size range of 0.6–4.75 mm, the proportion of the three grades of single-grade materials and grade ingredients of sand were determined by three repeated tests.

#### Single-grade material repeatability test

The results of the single-grade measurement in the range of 0.6–1.18 mm are listed in Table 4.

**Table 4:** Results of three tests of particle size of 0.6–1.18-mm sand (%).

Granularity (mm)	0.3–0.6	0.6–1.18	1.18–2.36	2.36–4.75
1	0.81	65.94	33.12	0.13
2	0.90	66.98	31.95	0.17
3	0.83	66.60	32.57	0
Δ	0.09	1.04	1.17	0.04

Table 4 indicates that, because of the different measurement principles, the screening method theoretically occupies 100% of the range of 0.6–1.18 mm, and the measured mass ratio of the self-developed image method occupies about 66%, with about 32% of the sand being 1.18–2.36 mm. For the three measurements of the same batch of samples, measured for a range of 0.6–1.18 mm, the repeatability error was 1.04%. The repeatability error of the tested sand was 1.17% for 1.18–2.36 mm.

The results of the single-grade measurement for a particle-size range of 1.18–2.36 mm are listed in Table 5.

**Table 5:** Results of three tests of particle size of 1.18–2.36 mm (%).

Granularity (mm)	0.6–1.18	1.18–2.36	2.36–4.75	4.75–9.5
1	0.56	64.46	34.89	0.09
2	0.20	61.45	38.35	0
3	0.41	64.19	35.40	0
Δ	0.36	3.01	3.46	0.09

Table 5 indicates that the repeatability error of the sand for the three test machines was 3.01% for a range of 1.18–2.36 mm. The maximum repeatability error was in a range of 2.36–4.75 mm, which is equal to 3.46%.

The results of the single-stage measurement for the particle-size range of 2.36–4.75 mm are shown in Table 6.

**Table 6:** Results of three tests of particle size of 2.36–4.75 mm (%).

Granularity (mm)	0.6–1.18	1.18–2.36	2.36–4.75	4.75–9.5
1	0.01	0.89	97.10	2.00
2	0.01	0.89	96.69	2.41
3	0.01	0.50	95.81	3.68
Δ	0	0.49	1.29	1.68

Table 6 shows that the mass ratio of the three trials for a range of 2.36–4.75 mm is 96% with the sieving method, while the repeatability error is 1.29%, The error is less than 1.18 to 2.36 mm for a single grade. However, the repeatability error of 4.75–9.5 mm is 1.68%, and the reproducibility of the system for 2.36–4.75-mm single-grade basically satisfies the engineering requirements.

Comparing the reproducibility of single-grade particles with sizes of 1.18–2.36 mm, the reproducibility of 0.6–1.18 mm appears to be optimum.

### Grade batching repeatability test

The results of the experiments using the prepared grading samples are listed in Table 7.

**Table 7:** Sand grading material test results (%).

Granularity (mm)	0.3–0.6	0.6–1.18	1.18–2.36	2.36–4.75	4.75–9.5
1	0.29	17.09	21.99	58.11	2.52
2	0.32	17.06	22.11	58.07	2.44
3	0.31	17.09	21.76	58.58	2.26
Δ	0.03	0.03	0.35	0.51	0.26

Table 7 shows that the results obtained for the three particle sizes are very close, with the maximum error in the 2.36–4.75-mm range being only 0.51%, while those for the other grain sizes being ≤ 0.35%.

### Contrasting with sieve method

#### Image based sieve method

Four manufactured sands produced from limestone and granite were used. These were identified as A, B, C, and D. A is granite form Fujian province; B is limestone form Fujian province; C is limestone form Hebei province; D is basalt form Hainan province. The four kinds of single sand materials were screened out from 0.6–1.18 mm, 1.18–2.36 mm, and 2.36–4.75 mm, respectively. Each single-grade material was loaded into the manufactured sand particle size detection system to detect its particle size distribution. The test results are listed in Tables 8–10.

**Table 8:** Image-based test results for four types of 0.6–1.18-mm sand (%).

Granularity (mm)	0.3–0.6	0.6–1.18	1.18–2.36	2.36–4.75
A	0	2.06	67.78	30.16
B	0.03	3.98	66.53	29.46
C	0.03	0.30	64.23	35.44
D	0.04	2.09	63.28	34.59

**Table 9:** Image-based test results for four types of 1.18–2.36-mm sand (%).

Granularity(mm)	0.6–1.18	1.18–2.36	2.36–4.75	4.75–9.5
A	0	2.06	67.78	30.16
B	0.03	3.98	66.53	29.46
C	0.03	0.30	64.23	35.44
D	0.04	2.09	63.28	34.59

**Table 10:** Image-based test results for four types of 2.36–4.75-mm sand (%).

Granularity (mm)	0.6–1.18	1.18–2.36	2.36–4.75	4.75–9.5
A	0	5.11	94.89	0
B	0	0.83	96.15	3.02
C	0	1.66	90.05	8.29
D	0	0	90.24	9.76

The results obtained for the three particle-size ranges of four types of manufactured sand show that, relative to the screening method, the results obtained with the image-based method are generally larger. The manufactured sand result for the 2.36–4.75-mm range is closest to that obtained for the screening method, reaching > 90%. The results obtained with all four methods are very close to each other. The results show that the maximum error is A and D at 1.18–2.36 mm, accounting for 58.47% and 66.67%, respectively. The reason for the error lies in the fact that the shape of sand D is relatively slender, while the difference between the particle size measured by the image-based method and that measured by the sieving method is small.

#### Image based grading sieve division method

Three kinds of single-graded materials were screened by the vibrating-sieve method. Three different kinds of ingredients were prepared in a range of proportions. The three kinds of ingredients were tested using the image-based method. The results are listed in Table 11. Here, Δ is the difference between the two results.

**Table 11:** Comparison of three different grades of ingredients as measured by sieve and image-based methods (%).

Granularity (mm)	Method	0.3–0.6	0.6–1.18	1.18–2.36	2.36–4.75
1	Sieve	0	16.67	33.33	50
	Image	0.28	11.63	23.93	64.16
	Δ	−0.28	5.04	9.37	-−14.16
2	Sieve	0	20	40	40
	Image	0.52	14.10	33.71	51.67
	Δ	−0.52	5.9	6.29	−11.67
3	Sieve	0	16.67	16.67	66.66
	Image	0.88	8.68	14.65	75.78
	Δ	−0.88	7.99	2.02	−9.11

Table 11 shows that the results obtained with the image-based method are greater than those obtained with the sieving method, and the results are consistent with the trend of single-graded material detection. This is due to the "minimum diameter" (the side dimension of the particles measured by the sieve size) of the particles as measured by the sieving method being less than the equivalent elliptical Feret short diameter used in the image measurement.

#### Result corrections

To ensure that the image-based method satisfies the requirements of the vibration-screening method, it is necessary to amend the image detection results. The correction algorithm is given as Eq (14):

$$D_{\text{pass}}=C\times D_{F_{\text{min}}}$$ (14)

**Fig 8:**
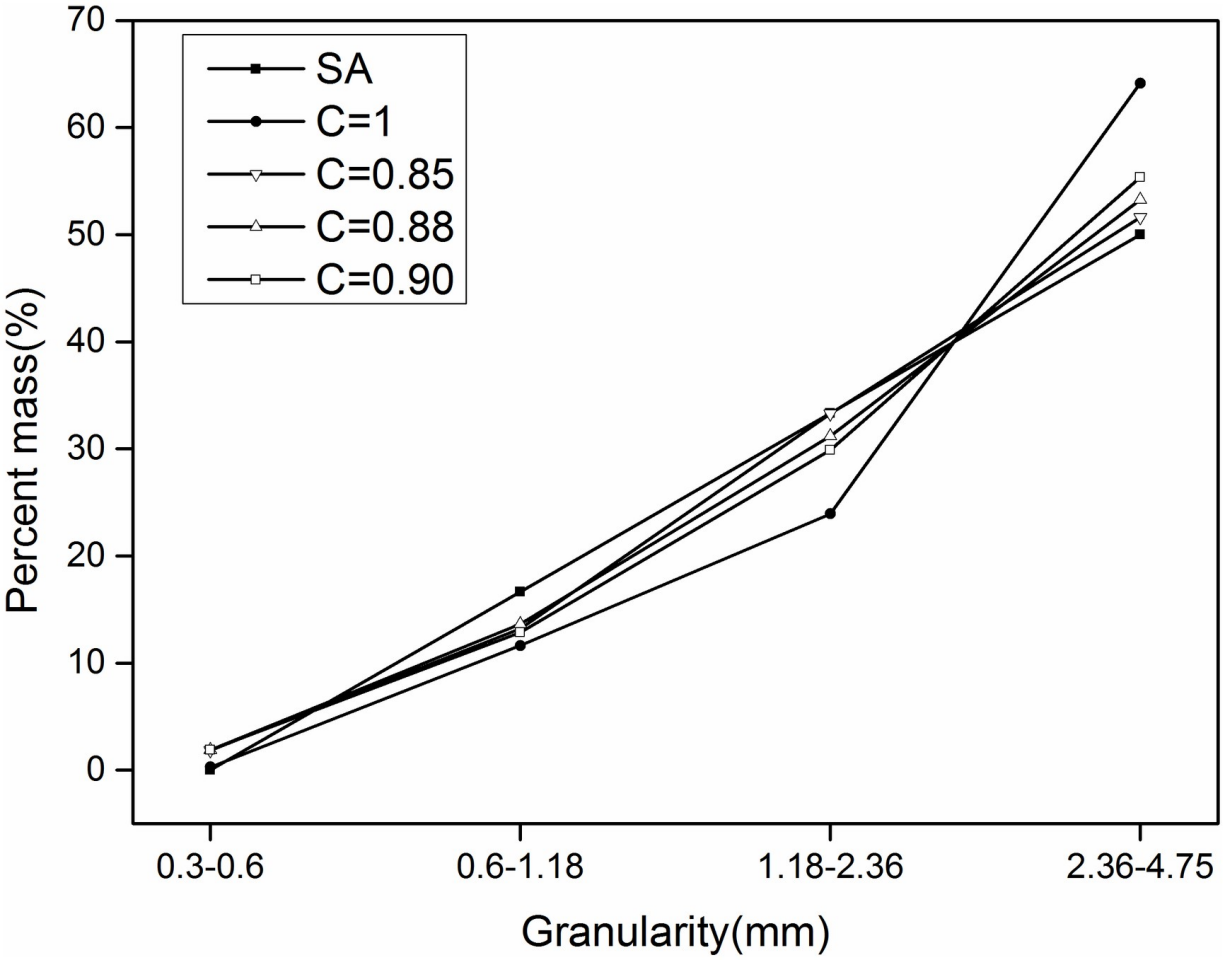
**Sand 1**: Comparison before and after correction (SA is sieve analysis).

**Fig 9:**
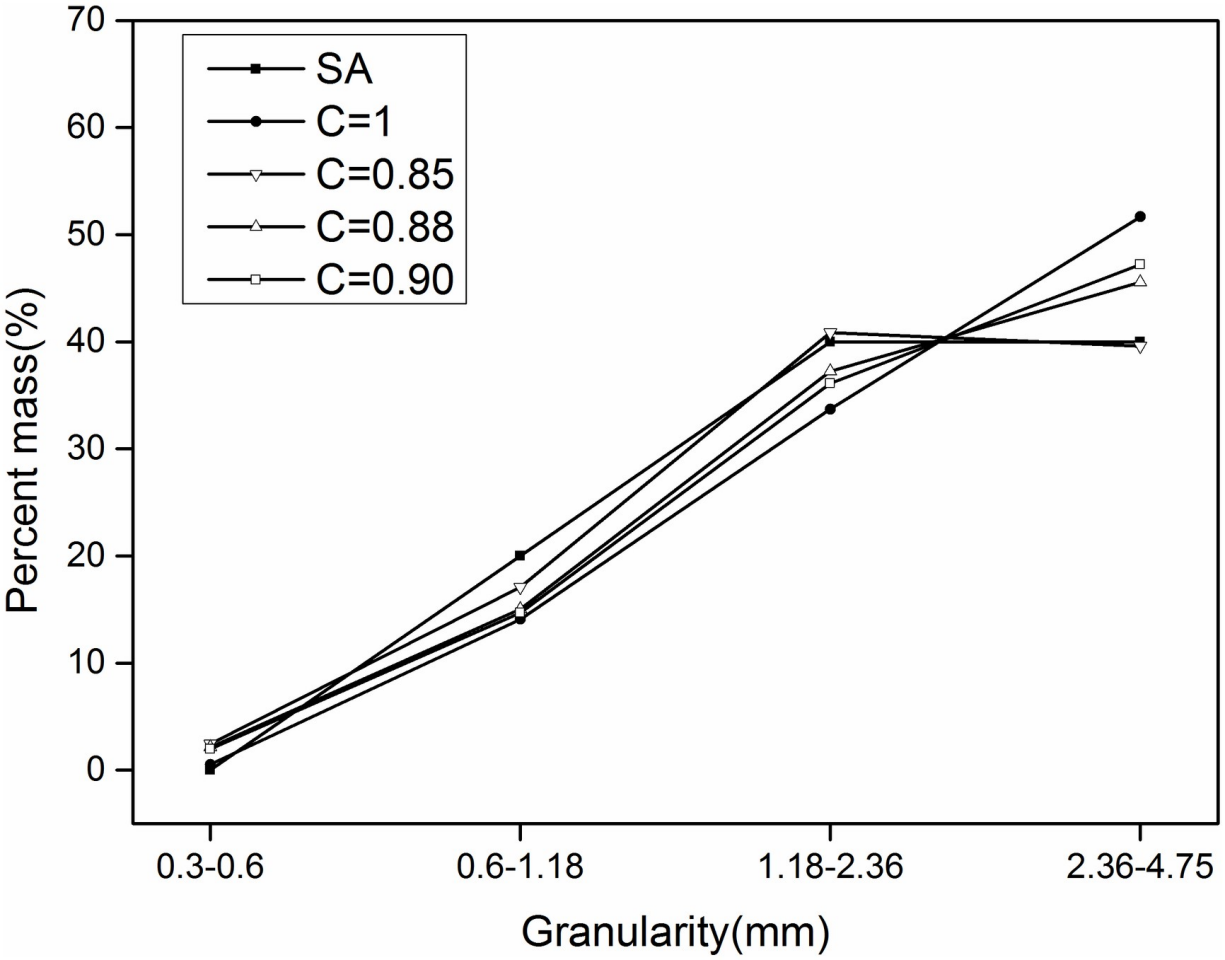
**Sand 2**: Comparison before and after correction (SA is sieve analysis).

**Fig 10:**
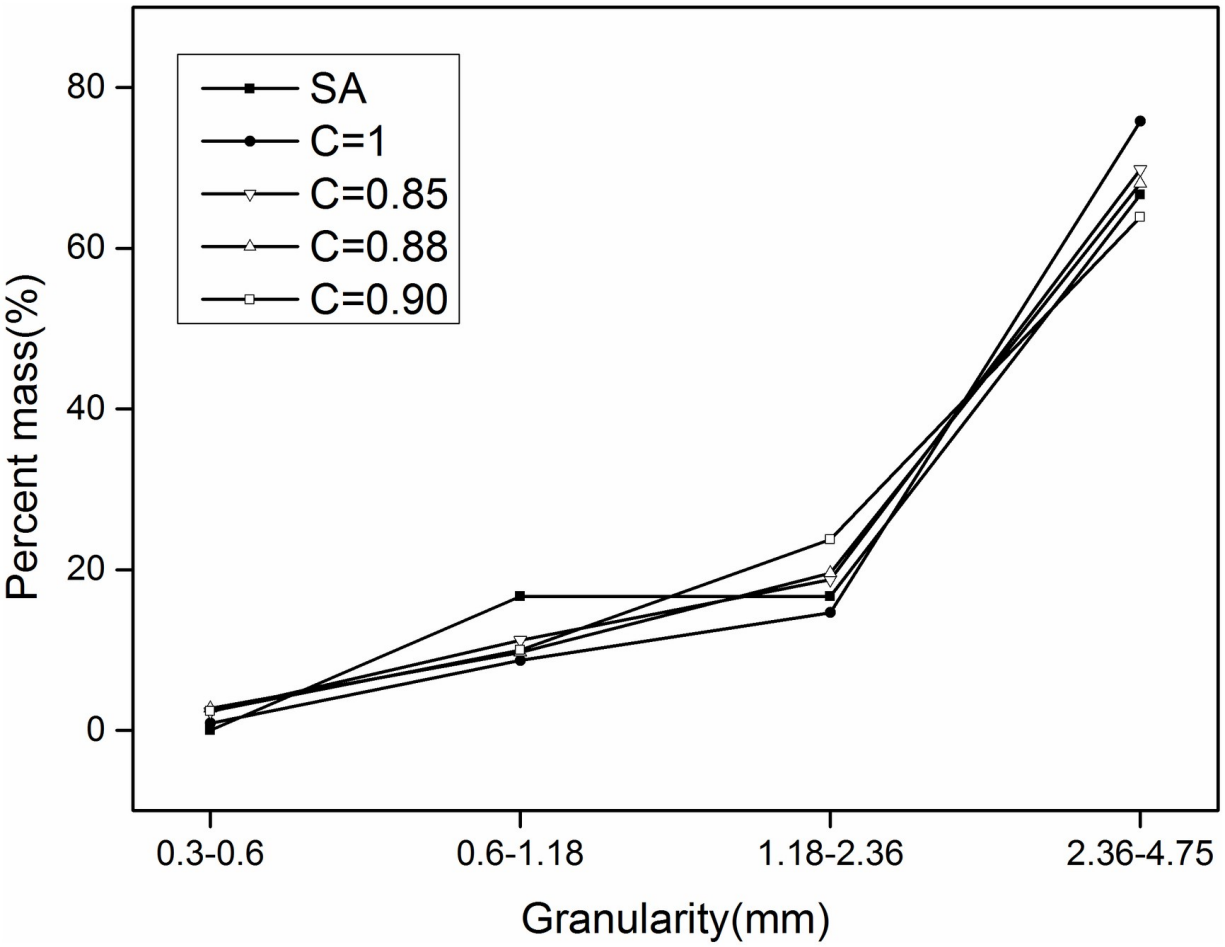
**Sand 3**: Comparison before and after correction (SA is sieve analysis).

Where D_pass_ is the smallest diameter, C is the conversion coefficient between the two paths, and D_Fmin_ is the equivalent ellipse Feret short diameter. The correction coefficient C was revised with the three values of 0.85, 0.88, and 0.9. The revised results are shown in Figs 8–10.

Fig 8 shows that, for the first-grade ingredients, when the correction factor is 0.85, the correction is better than 0.88 and 0.9. For the range of 1.18–2.36 mm, the correction result is 0.85 and the difference between the screening results and the sieving results is basically the same.

Fig 9 shows that, when the correction coefficient is 0.85, for size ranges of 1.18–2.36 mm and 2.36–4.75 mm, the corrected results are basically consistent with the results obtained with the screening method. For the range of 0.6–1.18 mm, the corrected results are similar to those obtained by sieving.

Fig 10 shows that the effect of the three correction coefficients on the results is not significant for the third batch with a size range of 0.6–1.18 mm. For a range of 1.18–2.36 mm, a correction factor of 0.85 is required to make the results obtained with the image-based method coincide with those obtained with the sieving method. For a range of 2.36–4.75 mm, the correction result is 0.88, which is the closest to that obtained with the sieving method.

Therefore, when the correction coefficient is 0.85, the results obtained with the image-based method and sieving method are in good agreement.

Table 12 lists the results obtained for the sand particle-size distribution after applying a correction factor of 0.85. Compared this with Table 11 reveals that the average error for the grade ingredients decreased from 7.22%, 6.10%, and 5% to 1.47%, 1.65%, and 3.23%, respectively.

**Table 12:** Comparison of results obtained with sieving method and image-based method (C = 0.85) (%).

Granularity (mm)		0.3–0.6	0.6–1.18	1.18–2.36	2.36–4.75
1	Sieve	0	16.67	33.33	50
	C = 0.85	1.85	13.23	33.28	51.62
	Δ	−1.85	3.44	0.05	−1.62
2	Sieve	0	20	40	40
	C = 0.85	2.45	17.08	40.85	39.61
	Δ	−2.45	2.92	−0.85	0.39
3	Sieve	0	16.67	16.67	66.66
	C = 0.85	2.3	11.2	18.73	69.77
	Δ	−2.3	5.47	−2.06	−3.11

## Conclusions

In the present study, a new method of measuring the size of sand grains was developed. The results were as follows:

1. A means of dispersing sand was designed and developed to be capable of fully dispersing falling sand. Experiment proved the uniformity and stability of sand falling.
2. The reproducibility of the sand granularity measurement system was studied experimentally. The repeatabilities of the granularity measurement systems were found to be 3.46% and 0.51%, respectively. The repeatability of the particle size detection was affected by the shape of the sand grains. When the content is high, the particle size measurement is less reproducible.
3. Comparative experiments show that the particle size as determined by the image-based method is larger than that determined by the sieving method. The results were therefore corrected by applying Feret’s correction coefficient. The error of the three methods is thus reduced from 7.22%, 6.10%, and 5% to 1.47%, 1.65%, and 3.23%, respectively.

This system achieves non-contact measurement of the manufactured sand without damaging the particles. It is faster and cheaper than conventional methods. This system can also be applied to different material particles, not limited to manufactured sand. In terms of particle size, the structure of the system’s scattered tube should be improved to meet the measurement of particles larger than 4.75mm. For particles less than 0.6mm, the manufactured sand transfer module and dispersion module of the system should be improved and optimized to facilitate the transportation and dispersion of particles to reduce the measurement error. In future, we believe this system will play an important role in aggregate quality assurance in many fields.

## Supporting information

S1 File The specific values of the data points in Figs 8–10. (XLSX)
